# Online information seeking by patients with bipolar disorder: results from an international multisite survey

**DOI:** 10.1186/s40345-016-0058-0

**Published:** 2016-08-24

**Authors:** Jörn Conell, Rita Bauer, Tasha Glenn, Martin Alda, Raffaella Ardau, Bernhard T. Baune, Michael Berk, Yuly Bersudsky, Amy Bilderbeck, Alberto Bocchetta, Letizia Bossini, Angela Marianne Paredes Castro, Eric Yat Wo Cheung, Caterina Chillotti, Sabine Choppin, Maria Del Zompo, Rodrigo Dias, Seetal Dodd, Anne Duffy, Bruno Etain, Andrea Fagiolini, Julie Garnham, John Geddes, Jonas Gildebro, Ana Gonzalez-Pinto, Guy M. Goodwin, Paul Grof, Hirohiko Harima, Stefanie Hassel, Chantal Henry, Diego Hidalgo-Mazzei, Vaisnvy Kapur, Girish Kunigiri, Beny Lafer, Chun Lam, Erik Roj Larsen, Ute Lewitzka, Rasmus Licht, Anne Hvenegaard Lund, Blazej Misiak, Patryk Piotrowski, Scott Monteith, Rodrigo Munoz, Takako Nakanotani, René E. Nielsen, Claire O’Donovan, Yasushi Okamura, Yamima Osher, Andreas Reif, Philipp Ritter, Janusz K. Rybakowski, Kemal Sagduyu, Brett Sawchuk, Elon Schwartz, Ângela Miranda Scippa, Claire Slaney, Ahmad Hatim Sulaiman, Kirsi Suominen, Aleksandra Suwalska, Peter Tam, Yoshitaka Tatebayashi, Leonardo Tondo, Eduard Vieta, Maj Vinberg, Biju Viswanath, Julia Volkert, Mark Zetin, Iñaki Zorrilla, Peter C. Whybrow, Michael Bauer

**Affiliations:** 1 Department of Psychiatry and Psychotherapy, University Hospital Carl Gustav Carus, Technische Universität Dresden, Dresden, Germany; 2 ChronoRecord Association, Fullerton, CA USA; 3 Department of Psychiatry, Dalhousie University, Halifax, Nova Scotia Canada; 4 Unit of Clinical Pharmacology, University Hospital of Cagliari, Cagliari, Italy; 5 Discipline of Psychiatry, School of Medicine, University of Adelaide, Adelaide, SA Australia; 6 IMPACT Strategic Research Centre, School of Medicine, Deakin University, Geelong, VIC Australia; 7 University Hospital Geelong, Barwon Health, Geelong, VIC Australia; 8 Department of Psychiatry, The University of Melbourne, Parkville, VIC Australia; 9 Florey Institute of Neuroscience and Mental Health, Parkville, VIC Australia; 10 Orygen Youth Health Research Centre, Parkville, VIC Australia; 11 Department of Psychiatry, Faculty of Health Sciences, Ben Gurion University of the Negev; Beer Sheva Mental Health Center, Beer Sheva, Israel; 12 Department of Psychiatry, University of Oxford, Warneford Hospital, Oxford, UK; 13 Section of Neurosciences and Clinical Pharmacology, Department of Biomedical Sciences, University of Cagliari, Sardinia, Italy; 14 Department of Molecular Medicine, Department of Mental Health (DAI), University of Siena, University of Siena Medical Center (AOUS), Siena, Italy; 15 Department of General Adult Psychiatry, Castle Peak Hospital, Hong Kong, China; 16 AP–HP, Hôpitaux Universitaires Henri-Mondor, Créteil, France; 17 Bipolar Disorder Research Program, Department of Psychiatry, University of São Paulo Medical School, São Paulo, Brazil; 18 Department of Psychiatry, University of Calgary, Calgary, Canada; 19 AP–HP, Hôpitaux Universitaires Henri-Mondor, INSERM U955 (IMRB), Université Paris Est, Créteil, France; 20 Department of Affective Disorders, Q, Mood Disorders Research Unit, Aarhus University Hospital, Aarhus, Denmark; 21 Department of Psychiatry, University Hospital of Alava, University of the Basque Country, CIBERSAM, Vitoria, Spain; 22 Mood Disorders Center of Ottawa, Ottawa, Canada; 23 Department of Psychiatry, University of Toronto, Ontario, Canada; 24 Department of Psychiatry, Tokyo Metropolitan Matsuzawa Hospital, Setagaya, Tokyo Japan; 25 Department of Psychology & Aston Brain Centre, Aston University, Birmingham, UK; 26 Institut Pasteur, Unité Perception et Mémoire, F-75015 Paris, France; 27 Bipolar Disorders Program, Hospital Clinic, University of Barcelona, IDIBAPS, CIBERSAM, Barcelona, Catalonia Spain; 28 Department of Clinical Psychology, NIMHANS, Bangalore, 560029 India; 29 Leicestershire Partnership NHS Trust, Leicester, UK; 30 Department of Psychiatry, Kowloon Hospital, Hong Kong, China; 31 Aalborg University Hospital, Psychiatry, Aalborg, Denmark; 32 Department of Clinical Medicine, Aalborg University, Aalborg, Denmark; 33 Department of Psychiatry, Wroclaw Medical University, Wroclaw, Poland; 34 Michigan State University College of Human Medicine, Traverse City Campus, Traverse City, MI USA; 35 Department of Psychiatry, University of California San Diego, San Diego, CA USA; 36 Affective Disorders Research Project, Tokyo Metropolitan Institute of Medical Science, Setagaya, Tokyo Japan; 37 Department of Psychiatry, Psychosomatic Medicine and Psychotherapy, University Hospital Frankfurt, Goethe-University, Frankfurt am Main, Germany; 38 Department of Adult Psychiatry, Poznan University of Medical Sciences,, Poznan, Poland; 39 Department of Psychiatry, University of Missouri Kansas City School of Medicine, Kansas City, MO USA; 40 Croton on Hudson, NY USA; 41 Department of Neuroscience and Mental Health, Federal University of Bahia, Salvador, Brazil; 42 Department of Psychological Medicine, Faculty of Medicine, University of Malaya, Kuala Lumpur, Malaysia; 43 City of Helsinki, Department of Social Services and Health Care, Psychiatry, Helsinki, Finland; 44 Department of Psychiatry, Department of Medicine, University of Hong Kong, Hong Kong, China; 45 Harvard Medical School–McLean Hospital, Boston, MA USA; 46 Lucio Bini Center, Cagliari e Roma, Italy; 47 Psychiatric Center Copenhagen, Copenhagen, Denmark; 48 Department of Psychiatry, NIMHANS, Bangalore, 560029 India; 49 Department of Psychology, Chapman University, Orange, CA USA; 50 Department of Psychiatry and Biobehavioral Sciences, Semel Institute for Neuroscience and Human Behavior, University of California Los Angeles (UCLA), Los Angeles, CA USA

## Abstract

**Background:**

Information seeking is an important coping mechanism for dealing with chronic illness. Despite a growing number of mental health websites, there is little understanding of how patients with bipolar disorder use the Internet to seek information.

**Methods:**

A 39 question, paper-based, anonymous survey, translated into 12 languages, was completed by 1222 patients in 17 countries as a convenience sample between March 2014 and January 2016. All patients had a diagnosis of bipolar disorder from a psychiatrist. Data were analyzed using descriptive statistics and generalized estimating equations to account for correlated data.

**Results:**

976 (81 % of 1212 valid responses) of the patients used the Internet, and of these 750 (77 %) looked for information on bipolar disorder. When looking online for information, 89 % used a computer rather than a smartphone, and 79 % started with a general search engine. The primary reasons for searching were drug side effects (51 %), to learn anonymously (43 %), and for help coping (39 %). About 1/3 rated their search skills as expert, and 2/3 as basic or intermediate. 59 % preferred a website on mental illness and 33 % preferred Wikipedia. Only 20 % read or participated in online support groups. Most patients (62 %) searched a couple times a year. Online information seeking helped about 2/3 to cope (41 % of the entire sample). About 2/3 did not discuss Internet findings with their doctor.

**Conclusion:**

Online information seeking helps many patients to cope although alternative information sources remain important. Most patients do not discuss Internet findings with their doctor, and concern remains about the quality of online information especially related to prescription drugs. Patients may not rate search skills accurately, and may not understand limitations of online privacy. More patient education about online information searching is needed and physicians should recommend a few high quality websites.

**Electronic supplementary material:**

The online version of this article (doi:10.1186/s40345-016-0058-0) contains supplementary material, which is available to authorized users.

## Background

Information seeking is an important aspect of coping with chronic illness (Lambert and Loiselle 2007; Brashers et al. 2002) and many patients with bipolar disorder want to learn more (Hallett et al. 2013; Giacco et al. 2014). The majority of patients with bipolar disorder would prefer to learn about their illness through a face-to-face conversation with their physician, as do patients with other mental and physical illness (Hallett et al. 2013; Gaglio et al. 2012; Hesse et al. 2005; Horgan and Sweeney 2010). This is often not feasible due to the large number of questions that will arise over the long term and the limited access to the treating psychiatrist. Due to the unique properties of the Internet, both patients and providers view it as an important source of all medical information, including information about bipolar disorder. The Internet is available globally, can be accessed on the patient’s schedule and content can be read at the patient’s pace (Korp 2006). Another unique aspect is that healthcare web sites are often viewed by patients in many countries (Leon and Fontelo 2016; Heilman and West 2015). For healthcare providers, large numbers of patients can be reached at a reasonable cost with a website.

The goal of this study was to better understand online information seeking by patients with bipolar disorder. This was investigated using an anonymous patient survey completed by 1222 patients with bipolar disorder in 17 countries. Our initial findings, reported previously, were that the patients used the Internet at a percentage that was similar to the general public (81 %), and that 78 % of the Internet users looked online for information on bipolar disorder or 63 % of the total sample (Bauer et al. 2016). The patients who looked online for information also consulted medical professionals plus other information sources, such as printed media, physician handouts, television, and other patients with bipolar disorder. This analysis will focus on the patterns of online information seeking, including why and how patients with bipolar disorder find information online, what information they are looking for, and the impact of information seeking.

## Methods

An anonymous, one-time survey was completed by patients with bipolar disorder. To maximize participation and minimize bias, the survey was paper-based and was translated into the local language. With a paper-based survey, patients without Internet skills or online access could be included. The treating psychiatrist provided the diagnosis, age of onset, and years of education for each patient. This study was approved by institutional review boards in accordance with local requirements.

### Survey

The survey contained 39 questions and took about 20 min to complete. The complete survey in English is available in the Additional file 1. The survey topics included questions about demographics, living with bipolar disorder, Internet use, online information seeking, and participation in online support groups. Questions 21–35 about online information seeking were analyzed here. The questions on Internet access were analyzed previously (Bauer et al. 2016). The survey was translated into 12 local languages: Chinese, Danish, Finnish, French, German, Hebrew, Italian, Japanese, Polish, Portuguese, Spanish, and English (versions for US/Canada, UK and Australia). A convenience sample was collected between March 2014 and January 2016. In total, 1222 surveys were received: from Australia (*N* = 22), Brazil (*N* = 100), Canada (*N* = 109), Denmark (*N* = 209), Finland (*N* = 16), France (*N* = 50), Germany (*N* = 82), Hong Kong (*N* = 91), India (*N* = 30), Israel (*N* = 46), Italy (*N* = 80), Japan (*N* = 35), Malaysia (*N* = 25), Poland (*N* = 125), Spain (*N* = 82), UK (*N* = 50), and the US (*N* = 70). The treatment settings included private practice, university clinics, and community mental health centers.

Duplicate data entry was used for quality control with the paper-based surveys (Kawado et al. 2003; Neaton et al. 1990). Automated logic checking of numeric fields was implemented as appropriate. More details about the survey methodology and validation were published previously (Bauer et al. 2016).

### Country variables

Since this was an international sample, country specific variables as well as the individual survey responses were included in the analysis. The county specific variables include the mean years of education for adults age ≥25 years (UNESCO 2015), education ratio (for those age ≥25 years, patient years of education compared to the mean years of education for general population), telecommunications data, and Hofstede’s cultural dimensions (Hofstsede 2016). The Hofstede’s power distance index (PDI) is a measure of social inequality where a lower number means that societies are questioning authority and striving to equalize the distribution of power.

### Statistics

Explanatory models were estimated to gain insight into relationships among survey responses, external variables and demographics. The generalized estimating equation (GEE) statistical technique was selected to overcome the imbalance in the number of responses from the collection sites, and to account for the correlation in survey responses among the collection sites. The GEE models were estimated using a binomial distribution, independent working correlation matrix and a logit link function. Many individual and country specific variables were similar and correlated. The potential variables from univariate analyses that were significant at a level of 0.05 were entered into multivariate models, and the corrected quasi-likelihood independence model criterion was used to assist with multivariate model fitting (Pan 2001). The odds ratios and confidence intervals generated by the GEEs are reported.

Descriptive statistics are reported for demographic variables. Survey responses are also summarized as the mean value of the individual country means. SPSS version 23.0 was used for all analyses.

## Results

The survey was completed by 1222 patients, with 51 more surveys received since the first analysis (Bauer et al. 2016). With 1212 valid responses, 976 (81 %) patients used the Internet. Of the 972 valid responses, 750 (77 %) used the Internet to look up information about bipolar disorder or 61 % of the total sample. The demographics for all patients who completed the survey and for the patients who used the Internet to find information about bipolar disorder are shown in Table 1. The 750 patients who used the Internet to find information on bipolar disorder had a mean age of 41 years ± 12.5, were 62 % female, and had a mean of 14 ± 3.0 years of education.

**Table 1:** Patient demographics

Variable	Value	All patients (*N* = 1222)		Used Internet to find out about bipolar disorder (BP) (*N* = 750)	
		*N* ^a^	%	*N* ^a^	%
Diagnosis	BP I	768	63.7	440	59.7
	BP II	380	31.6	256	34.7
	BP NOS	57	4.7	41	5.6
Gender	Female	759	62.3	465	62.2
	Male	459	37.7	283	37.8
Area of residence	Urban	744	61.2	451	60.3
	Suburban	291	23.9	196	26.2
	Rural	181	14.9	101	13.5
Employment status	Full-time	560	46.6	396	53.6
	Not full-time	641	53.4	343	46.4
Marital status	Married	593	48.9	359	48.3
	Not married	619	51.1	385	51.7
Income group	Upper income	80	6.6	60	8.1
	Middle income	594	49.1	363	48.9
	Lower income	535	44.3	320	43.1
Live alone	Yes	299	24.7	190	25.5
	No	913	75.3	556	74.5
Mood in last six months	Mostly normal	581	47.9	317	42.6
	Mostly not normal	632	52.1	428	57.4
BP interfered with regular activities	Frequently or sometimes	768	63.2	524	70.1
	Rarely or never	448	36.8	223	29.9
Confident managing living	Very confident	454	37.5	259	34.8
	Not very confident	757	62.5	486	65.2
Confident when to see doctor about BP	Very confident	698	57.4	418	56.0
	Not very confident	518	42.6	328	44.0

Means		N^a^	Mean (SD)	N^a^	Mean (SD)
Age		1217	44.4 (13.8)	748	41.1 (12.5)
Years of education		1199	14.0 (3.2)	737	14.4 (3.0)
Age of onset		1201	27.1 (10.9)	734	25.6 (10.1)
Years of Illness		1194	17.4 (12.2)	730	15.6 (11.6)

^a^Missing values not included

### Survey responses

The most common responses to the questions are shown as the mean value of the individual country means in Table 2. When looking for information about bipolar disorder, 89 % used a computer and 11 % used a smartphone or tablet. When online, 79 % started looking with a general search engine. 69 % of the patients rated their Internet search skills as basic or intermediate, and 31 % as expert. When searching, 16 % indicated that they always find what they are looking for, 57 % most of the time, 19 % half of the time, 8 % less than half, and 0.3 % never. The primary reasons that patients searched included side effects from prescription drugs (51 %), to learn anonymously (43 %), and because they need help coping (39 %). 62 % of the patients looked for information a couple of times a year, and 38 % looked monthly or more frequently.

**Table 2:** Summary of responses from the patients who used the Internet to find information on bipolar disorder (*N* = 750)

Question number	Question^a^	Valid responses *N*	Variable	Mean percent^b^ (%)	SD^c^
21^d^	How do you access the Internet?	607	From a computer	89	0.1221
		607	From a smartphone or tablet	11	0.1221
22	How do you rate your Internet search skills?	746	Basic or intermediate	69	0.1288
		746	Expert	31	0.1288
23	How do you start looking for information about bipolar disorder?	623	General search engine	79	0.0816
		623	Medical search engine	6	0.0478
		623	Specific site on mental illness	10	0.0640
		623	Other	6	0.0475
24	Do you find what you are looking for?	733	Always	16	0.1094
		733	Most of the time	57	0.1277
		733	About half the time	19	0.0847
		733	Less than half the time	8	0.0865
		733	Never	0.3	0.0073
25	Do you look for these topics relating to bipolar disorder?^e^	742	Prescription drug information	73	0.1363
		742	Symptoms	83	0.1173
		742	General course of illness	58	0.1268
		742	Coping strategies	52	0.1774
26	Do you look for these topics relating to getting treatment for bipolar disorder?^e^	542	Clinic hours, location and directions	52	0.2079
		542	Physician or therapist credentials	31	0.1633
		542	Physician or therapist ratings	24	0.1711
27	How frequently do you search for information about bipolar disorder?^e^	738	Monthly or more	38	0.1620
		738	Couple of times a year	62	0.1620
28	Why do you search for information about bipolar disorder on the Internet?^e^	735	To learn anonymously	43	0.1600
		735	Side effects from prescription drugs	51	0.1184
		735	Need help coping with the illness	39	0.1706
29	What are your favorite sources of information about bipolar disorder?^e^	727	Specific sites on mental health or bipolar disorder	59	0.1744
		727	Online encyclopedia such as Wikipedia	33	0.0843
		727	Government sponsored sites	21	0.2168
30	Do you discuss online information about bipolar disorder with your doctor?	737	Sometimes or frequently	33	0.1178
		737	Rarely	67	0.1178
31	Does the online information help you cope with bipolar disorder?	733	Sometimes for frequently	67	0.1477
		733	Rarely	33	0.1477
32	Do you attempt to verify online information?	735	Yes	66	0.1158
33	How do you attempt to verify online information?^e,f^	428	Discuss with doctor	62	0.1633
		428	Discuss with family and friends	37	0.1536
		428	Compare information on multiple sites	56	0.1620
34	How concerned are you about privacy and confidentiality?	723	Moderately or very concerned	32	0.1788
		723	Slightly concerned	68	0.1788
35	Do you participate in patient support groups?	746	Yes	20	0.0883

^a^See survey for details and exact wording

^b^Overall mean of country means

^c^Standard deviation of country means

^d^Survey questions 1–16 on demographics; questions 17-20 analyzed previously (Bauer et al. 2016)

^e^More than one response allowed

^f^Only completed by those who attempt to verify information (*N* = 485)

The patients looked frequently for a variety of topics related to bipolar disorder including symptoms (83 %), prescription drug information (73 %), general course of illness (58 %), coping strategies (52 %), and side effects from prescription drugs (51 %). The patients also looked for topics related to the healthcare system, although the percentage of missing responses differed among the countries. The patients looked frequently for clinic hours, location and driving instructions (52 %), physician credentials (31 %) and physician ratings (24 %). Patients looked less frequently for all remaining responses. For favorite sources of information, 59 % indicated a specific site on mental health or bipolar disorder, 33 % indicated Wikipedia and 21 % a government sponsored site. 66 % of the patients said they attempted to verify the information found online, primarily by discussing with a doctor (62 %) or comparing information on multiple sites (56 %).

67 % of the patients rarely or never discussed what they found online with their doctors, while 33 % sometimes or frequently did. 67 % of the patients said that information seeking online helped them to cope sometimes or frequently, while 33 % said rarely or never. Only 32 % of the patients were moderately or very concerned about privacy while 68 % were slightly or not concerned. Only 20 % of the patients read or participated in online support groups.

### Explanatory models

The details for the explanatory models are shown in Table 3. To explain Internet skills self-rated as expert, the estimated coefficients from the best fitting model suggested that a 1 year increase in age will decrease the odds of having expert skills by 3 %, having Wikipedia as a favorite source will increase the odds by 49 %, attempting to verify the information found online will increase the odds by 72 %, being male will increase the odds by 89 %, and feeling very confident about when to see the doctor about bipolar disorder will increase the odds by 89 %.

**Table 3:** Explanatory models based on responses from the patients who used the Internet to find information on bipolar disorder (*N* = 750)

Question number	Dependent variable		*N*	Independent variables			
	Question^a^	Answer^b^		Parameter	Significance	OR	95 % CI
22	How do you rate your Internet search skills?	Expert	725	Intercept	0.010	0.463	0.257, 0.835
				Age	<0.001	0.970	0.956,0.984
				Male	0.004	1.892	1.221, 2.931
				Very confident when to see the doctor	<0.001	1.891	1.354, 2.639
				Favorite source Wikipedia	<0.001	1.487	1.221,1.812
				Attempt to verify the online information	0.003	1.717	1.198, 2.460
24	Do you find what you are looking for?	Always	661	Intercept	<0.001	0.203	0.127, 0.323
				Consult more than one medical professional (e.g., counselor or psychologist) plus psychiatrist	0.033	0.588	0.361, 0.958
27	How frequently do you search for information about bipolar disorder?	Monthly or more often	703	Intercept	0.006	0.310	0.135, 0.709
				Education ratio	0.004	0.621	0.448, 0.858
				Male	0.025	1.525	1.054, 2.207
				Mostly normal last 6 months	<0.001	0.395	0.262, 0.596
				Bipolar disorder interferes frequently or sometimes	<0.001	1.952	1.406, 2.711
				Search for side effects from prescription drugs	0.021	1.465	1.060, 2.024
				Favorite source are specific sites on mental health	0.021	1.292	1.039, 1.607
				Online information helps to cope	<0.001	2.699	2.008, 3.627
				Participate in online support groups	<0.001	2.286	1.552, 3.367
29	What are your favorite sources of information about bipolar disorder?	Specific sites on mental health or bipolar disorder	642	Intercept	<0.001	0.190	0.087, 0.415
				Age	0.001	1.022	1.009, 1.035
				Male	0.002	0.729	0.595, 0.892
				Consult more than one medical professional	0.001	1.515	1.197, 1.917
				Search monthly or more often	0.019	1.383	1.054, 1.814
				Online information helps to cope	<0.001	1.598	1.284, 1.990
				Attempt to verify the online information	<0.001	2.670	2.070, 3.445
29	What are your favorite sources of information about bipolar disorder?	Wikipedia	744	Intercept	<0.001	0.293	0.211, 0.406
				Male	0.006	1.565	1.135, 2.158
				Expert search skills	<0.001	1.579	1.271, 1.963
30	Do you discuss online information about bipolar disorder with your doctor?	Sometimes or frequently	715	Intercept	<0.001	0.068	0.038, 0.123
				Very confident when to see the doctor	0.001	1.936	1.297, 2.891
				Online information helps to cope	<0.001	2.621	1.827, 3.761
				Attempt to verify the online information	<0.001	3.149	2.374, 4.177
31	Does the online information help you cope with bipolar disorder?	Sometimes or frequently	711	Intercept	0.005	0.610	0.433, 0.861
				Mostly normal last 6 months	<0.001	2.015	1.525, 2.663
				Always find what looking for	<0.001	2.637	1.706, 4.076
				Search monthly or more often	<0.001	2.290	1.659, 3.160
				Online information helps to cope	<0.001	1.684	1.276, 2.221
				Discuss findings with doctor	<0.001	3.023	2.024, 4.517
34	How concerned are you about privacy and confidentiality?	Moderately or very concerned	723	Intercept	<0.001	0.085	0.049, 0.147
				Power Distance Index	<0.001	1.030	1.020, 1.040
				Search online to learn anonymously	0.001	1.705	1.260, 2.308

^a^See survey for details and exact wording

^b^Missing values not included

To explain if patients always find what they are looking for, the estimated coefficients from the best fitting model suggested that consulting another medical professional (e.g., primary care, psychologist or counselor) in addition to a psychiatrist will decrease the odds by 41 %. There was no association with expertise or education.

To explain if patients search monthly or more frequently, the estimated coefficients from the best fitting model suggested that a one unit increase in education ratio will decrease the odds by 38 %, having a mostly normal mood for the last 6 months will decrease the odds by 60 %, having a mental health site as a favorite source will increase the odds by 29 %, looking for drug side effects will increase the odds by 47 %, being male will increase the odds by 53 %, bipolar disorder interfering with life will increase the odds by 95 %, participating in online support groups will increase the odds by 129 %, and feeling the Internet helps to cope will increase the odds by 170 %.

There was no association between the favorite sources of online information and coming from a country where the primary language is English. To explain having a mental health site as a favorite source, the estimated coefficients from the best fitting model suggested that a 1 year increase in age will increase the odds by 2 %, being male will decrease the odds by 27 %, searching monthly or more frequently will increase the odds by 38 %, consulting another medical professional will increase the odds by 52 %, needing help coping will increase the odds by 60 %, and attempting to verify the information found online will increase the odds by 167 %. To explain having Wikipedia as a favorite source, the estimated coefficients from the best fitting model suggested that being male will increase the odds by 57 %, and having self-rated expert search skills will increase the odds by 58 %.

To explain if the patients discuss online information with their doctor, the estimated coefficients from the best fitting model suggested that feeling very confident when to see the doctor will increase the odds by 94 %, feeling the Internet helps to cope will increase the odds by 162 %, and attempting to verify the information found online will increase the odds by 215 %.

To explain if patients feel the information learned online helps to cope, the estimated coefficients from the best fitting model suggested that needing help coping will increase the odds by 68 %, having a mostly normal mood for the last 6 months will increase the odds by 102 %, searching monthly or more frequently will increase the odds by 129 %, always find what looking for will increase the odds by 164 %, and discussing findings with the doctor will increase the odds by 202 %.

To explain if patients are concerned about privacy, the estimated coefficients from the best fitting model suggested that a one unit increase in the country PDI will increase the odds by 3 %, and going online to learn anonymously will increase the odds by 71 %.

## Discussion

### Three reasons for online information seeking

There were three primary reasons why patients looked online for information about bipolar disorder: prescription drugs, perceived anonymity, and help coping. About half the patients looked online because of side effects from prescription drugs, in agreement with prior reports that patients with bipolar disorder want to know more about their drugs and especially side effects (Hallett et al. 2013; Bowskill et al. 2007). Many patients looked online to learn about bipolar disorder without revealing their identity. Although incorrect, the belief that one is anonymous online is a commonly perceived benefit of the Internet by those with a stigmatized illness (Berger et al. 2005; Pohjanoksa-Mäntylä et al. 2009; Chan et al. 2016). Patients also sought information online because they were having difficulty coping with the illness, confirming the need for online sources to help deal with the consequences of bipolar disorder.

### Using a general search engine on a computer

The vast majority of patients started looking for information on bipolar disorder from a general search engine (79 %) on a laptop/desktop computer (89 %). This is consistent with prior findings that 80 % of the general public started looking for health information with a general search engine (Fox and Duggan 2013), and that general search engines are used much less frequently from a smartphone than from a computer (Arthur 2015; MacMillan 2015; Friedman 2015). Providers involved in developing online projects for bipolar disorder should consider how the targeted consumer device may impact use. About 43 % of total handsets in the world were smartphones in 2015, with an expected increase to 50 % by 2020 (Cisco 2016). In some countries such as the US, those with mental illness may have a much lower smartphone ownership than the general public (Klee et al. 2016; Miller et al. 2016).

### General search engine background

Most patients start looking for information from a general search engine. Google dominates with a market share of ≥90 % in all but 3 of the 17 countries in this study, the exceptions being 87 % in Canada, 73 % in Hong Kong and 73 % in the US (Return on Now 2016). Search terms entered are brief and guided by auto-completion suggestions, with 57 % in Google US being 1–2 words, and 88 % being 4 words or less (Statistica 2016). Over 90 % of people select a website from the first page of search engine results, with 61 % of people selecting a web site from the top 3 results (Sharp 2014).

### 1/3 rated search skills as expert

In this study, about 2/3 of the patients rated their search skills as basic or intermediate, and 1/3 as expert. There was no association between education and self-rating as an expert. The factors that increased the odds of self-rating as an expert included being male, having confidence when to see the doctor about bipolar disorder, having Wikipedia as a favorite source, and attempting to verify the information. In prior research, self-reported computer skill ratings were found to be unreliable (Merritt et al. 2005), with university students tending to overestimate their abilities (Ivanitskaya et al. 2006), and females perceiving lower abilities than males at the same skill level (Bunz et al. 2007). There are several concerns about the accuracy of the self-rating of search expertise in this study.

A minority of patients (16 %) always found what they were looking for, but this was not associated with expertise, education or attempting to verify the information. However, always finding the answer was associated with not consulting more than one medical professional. There is concern that some patients may be satisfied with any answer, or use the Internet as a second opinion.

Although 1/3 of the patients rated their search skills as expert, it is quite difficult to search for medical information. In a recent study, no answer was found in the top three search results returned for 32 of 54 simple consumer mental health questions using the National Institute of Mental Health (NIMH) website (Crangle and Kart 2015). Patient questions are not answered due to a high degree of specificity, false presuppositions, and layman use of medical terminology (Crangle and Kart 2015; MacCray et al. 1999). In an analysis of consumer messages to MedlinePlus, the majority concerned specific medical questions rather than general information (Miller 2007). For perspective, physicians also report barriers to successful online searching including the need for specific information, too much information, selecting reliable sources, and time requirements (Clarke et al. 2013; Bennett et al. 2004; McKibbon and Fridsma 2006).

There was no association between concern about privacy and expertise. In this study, 43 % of patients believed they are anonymous online, a result similar to the 37 % found in a survey of the US general public (Rainie et al. 2013). Although the legal framework for online privacy varies internationally (Bowman 2016), this finding emphasizes an important need to educate patients at all levels of expertise about privacy on the Internet. Commercial firms that provide services at no charge, including search engines, social media, and many medical sites, generally make money by tracking all activities to sell targeted behavioral advertising, or by selling the tracked activities to third parties (Greengard 2012; Stark and Fins 2013; Glenn and Monteith 2014; FTC 2014; Eavis 2016; Rosenberg 2015). The data collected includes details about all Internet and smartphone activities including search terms, websites visited, email and social media content and metadata (data about data that provides context) (Libert 2015; NISO 2004). In a recent study of over 80,000 health-related websites in the US, over 90 % sent data to third parties, with 70 % including specific symptoms, treatments and diseases (Libert 2015). Those who use social media and online support groups owned by commercial organizations may not realize that privacy policies often give consent to the sale of patient created data (Li 2013; Lupton 2014; Glenn and Monteith 2014). Online medical data may be combined with other data for health risk predictions (Dixon and Gellman 2014), combined with genetic data for commercial research (Seife 2013), and de-identified data may be re-identified using very large, high-dimensional databases (Narayanan et al. 2016). Even though the legal framework is changing, such as with the general data protection regulation (GDPR) to be implemented by 2018 for the EU (European Parliament News 2016), patients of all search skill levels need to understand the data policies of websites they visit regularly for information on mental health.

### Favorite sources are mental health sites and Wikipedia

For 59 % of the patients in this study, a mental health site was a favorite information source on bipolar disorder, while Wikipedia was a favorite source for 33 %. The factors that increased the odds for a mental health site included being female, needing help coping, consulting more than one medical professional, and attempting to verify the information. Being male or having expert search skills increased the odds that Wikipedia was a favorite source.

### Types of mental health sites

Websites on mental health are owned by a variety of entities, including governments, professional organizations, and commercial businesses. The overall content of most Internet information related to affective disorders is generally rated as good (Grohol et al. 2014; Morel et al. 2008) although the quality may vary considerably (Reavley and Jorm 2011; Hasty et al. 2014; Barnes et al. 2009; Monteith et al. 2013). The content of government websites, such as from the US MedlinePlus and UK NHS are evaluated for accuracy and currency (MedlinePlus 2016; NHS 2016). Hospitals and health systems own many websites and understand the importance of accuracy, but also view these websites as marketing opportunities (Ford and Walls 2012). Website content from providers that offer specific treatments, such as substance abuse treatment centers, may be incomplete or imbalanced (Schenker and London 2015; Link et al. 2016).

Articles from Wikipedia, the open encyclopedia that invites user contributions, frequently appear in first page of results for medical searches (Laurent and Vickers 2009). The Wikipedia entry for bipolar disorder was viewed over 13 million times in 55 languages in 2013 (Heilman and West 2015). In 2013, there were less than 300 editors for the medical articles on Wikipedia, of which 82 % were male and 50 % were healthcare providers (Heilman and West 2015). About 85 % of the medical editors had a college degree, with editors of the non-English versions having a similar education level. As in this study, more males (56 %) than females (44 %) are readers of Wikipedia about all topics (Pande 2011).

As of 2015, the Google search engine returns pre-vetted facts at the top of the first page for many medical searches (Google 2015). Beyond this, results may be tailored to the user based on behavioral advertising algorithms (Monteith et al. 2013; Vaidhyanathan 2012). The results of medical searches from all major search engines overlap considerably (Wang et al. 2012).

### Website language

There was no association between a native language other than English and the favorite information source in this study. Based on the top 10 million websites on the Internet, 53.6 % of all content is in English (W3 Techs 2016). The percent of content in German, Japanese, Spanish and French ranged between 4 and 6 % each, with a smaller percent in the other languages in this study. One consequence of the Internet is a global increase in teaching English as a second language. In 2014, 77 % of primary school students in the EU learned English as a foreign language, up from 35 % in 2000 (Eurostat 2016; Parker 2015).

### Searching a couple times a year

Most of the patients in this study (62 %) looked online for information a couple of times a year, while 38 % looked monthly or more often. The odds of looking online more frequently were increased if bipolar disorder interfered with life, the patient was looking for drug side effects, felt the internet helps to cope, read or contributed to an online support group, or if male. The odds of looking online more frequently were decreased if patients were mostly euthymic, or if they were very educated. Both experiencing symptoms and participation in online support groups were previously noted to increase the frequency of information seeking (Rice 2006; Weaver et al. 2010). The very educated may prefer to consult with their doctor for individualized treatment (Bauer et al. 2016).

### Seeking information on medical topics

The patients in this study most often sought information about medical topics, including symptoms (83 %), prescription drug information (73 %), general course of illness (58 %), coping strategies (52 %), and less frequently about nonmedical topics including clinic location, hours and directions (52 %), and physician credentials (31 %).

The strong patient interest in prescription drug information is important since both physical and mental side effects are associated with nonadherence (Bates et al. 2010; Johnson et al. 2007; Baldessarini et al. 2008; Szmulewicz et al. 2016). The accuracy of drug information on websites for consumers is of concern. Research has focused on Wikipedia, and compared the content for a variety of drugs to official product labels or professionally edited compendia. The drug information content on Wikipedia was mostly accurate but lacked details, was often incomplete (Kupferberg and Protus 2011; Clauson et al. 2008; Lavsa et al. 2011), and drug safety information may not be current (Koppen et al. 2015; Hwang et al. 2014). Some drug articles on Wikipedia were missing references (Lavsa et al. 2011) and unlike professional compendia, cited news stories as references (Koppen et al. 2015). Similarly, studies of accuracy of drug information on other consumer websites found generally good content quality but incomplete topic coverage (Sidhu et al. 2006; Ghoshal and Walji 2006).

Drug review websites based on patient ratings of drugs are also available online. Unlike standard compendia, patient review sites emphasize side effects and personal ratings of efficacy. These sites may over-represent the experiences of patients with acute exacerbations of illness, severe side effects or dissatisfaction with results (Chew and Khoo 2016; Hughes and Cohen 2011). Comparisons of drug efficacy ratings found that results varied among the patient review sites (Gidwani and Zulman 2015; Chew and Khoo 2016).

Patient education should emphasize that online drug information may be inaccurate or incomplete, even in compendia (Randhawa et al. 2016), and that patients should seek multiple sources. The emphasis on personal experience in drug review sites may be useful to some patients (Hughes and Cohen 2011; Chew and Khoo 2016), but patient education should also explain that these sites are not authoritative. Some patients may not realize that high quality drug information is available for free from government sources such as the US National Library of Medicine Drug Information Portal (NLM 2016).

### Information seeking helps with coping

Online information seeking sometimes or frequently helped 67 % of the patients in this study to cope. In this study, the factors that increased the odds that information seeking helped to cope included being mostly euthymic, always finding what looking for, searching monthly or more frequently, needing help coping, and discussing findings with the doctor. In a prior study of high functioning patients with bipolar disorder, education about the disease was a key strategy for staying well (Murray et al. 2011).

Only 20 % of the patients read or participated in online support groups or forums, a rate similar to that for the general public (Fox 2011). In this study, participation in online support groups was not associated with information seeking helping patients to cope. This finding suggests that the information learned online is what helps most patients to cope, rather than emotional support or coping skills learned in online support groups. Prior research on online communities reported that information seeking was the most important activity, (Nambisan 2011; Meier et al. 2007; van Uden-Kraan 2009), although emotional support was also important in bipolar disorder (Bauer et al. 2013). The time length of most online psychological interventions is considerably shorter than for face-to-face programs and may not offer sufficient professional access for patients to learn coping skills (Hidalgo-Mazzei et al. 2015).

In this study, 67 % of the patients rarely or never discussed what they learned online with their doctor. About 60–80 % of general medical patients also do not discuss Internet health information with their doctor for reasons including time constraints, fear of insulting the doctor or being dismissed, information was self-explanatory, and a desire for self-management (Chung 2013; Russ et al. 2011; Hay et al. 2008; Kim and Kim 2009; Diaz et al. 2002; Imes et al. 2008). In this study, the factors that increased the odds of patients discussing online information with their doctor include feeling very confident about when to see the doctor, feeling the Internet helped to cope, and attempting to verify the information. Similarly, in prior research, patients who talked with their doctors felt the information from the Internet was of high quality (Diaz et al. 2002; Chung 2013).

The patients in this study had a mean of 17 years of illness, so it is likely that many searches were for specific questions rather than general information about bipolar disorder. Since most patients do not discuss the information learned online with their doctor, patient education about online health information seeking is important, and should be integrated into psychoeducation programs. There is a need to help patients to access high quality information, such as by providing a short list of recommended websites (Monteith et al. 2013). Website recommendations from physicians are favorably received by general medicine patients, and may increase communication about online information seeking (Coberly et al. 2010; Siegel et al. 2006). Patients should also understand that even a clear and accurate website will probably not provide answers to specific questions about their personal situation (Miller 2007).

## Limitations

Since this survey was collected as a convenience sample, the survey participants do not reflect the demographic compositions of the countries which may bias the results. There was no follow-up discussion of the survey responses, and patient actual use of the Internet could not be verified. The patients not included in this study are those with bipolar disorder who do not seek professional help, who may be less educated or have a less stable living situation. Valid response rates varied among the questions analyzed. However, all results were consistent with prior research. These survey results are associations and do not establish causality between variables. This survey was administered by the treating psychiatrist so questions on patient satisfaction were omitted, although dissatisfaction is associated with increased online searching for health information (Tustin 2010). The survey did not address if patients visited sites in a language other than their native language or which specific websites were visited. The survey also did not address the use of problematic websites that encourage unwanted behaviors such as self-harm (Mitchell et al. 2014), issues related to online purchase of prescription or illegal drugs (Monteith et al. 2016; Orizio et al. 2011), or the impacts of direct to consumer pharmaceutical advertising where applicable (Mackey et al. 2015).

## Conclusions

Online information seeking helped two thirds of the patients to cope with bipolar disorder. When considering all 1222 patients who completed the survey, about 41 % of all patients found help coping using the Internet, so other information sources remain important. Most patients do not discuss information learned online with their doctor. There is concern about the quality of website content especially related to prescription drugs. Patients may not rate search skills accurately, and may not understand online privacy issues. More patient education about online information seeking is needed, and physicians should recommend a few high quality websites about bipolar disorder to their patients.

## Additional file

**Additional file 1.** Questionnaire: Information seeking in bipolar disorder.
