# Erratum to: Online information seeking by patients with bipolar disorder: results from an international multisite survey

**DOI:** 10.1186/s40345-017-0082-8

**Published:** 2017-03-31

**Authors:** Jörn Conell, Rita Bauer, Tasha Glenn, Martin Alda, Raffaella Ardau, Bernhard T. Baune, Michael Berk, Yuly Bersudsky, Amy Bilderbeck, Alberto Bocchetta, Letizia Bossini, Angela Marianne Paredes Castro, Eric Yat Wo Cheung, Caterina Chillotti, Sabine Choppin, Maria Del Zompo, Rodrigo Dias, Seetal Dodd, Anne Duffy, Bruno Etain, Andrea Fagiolini, Julie Garnham, John Geddes, Jonas Gildebro, Ana Gonzalez-Pinto, Guy M. Goodwin, Paul Grof, Hirohiko Harima, Stefanie Hassel, Chantal Henry, Diego Hidalgo-Mazzei, Vaisnvy Kapur, Girish Kunigiri, Beny Lafer, Chun Lam, Erik Roj Larsen, Ute Lewitzka, Rasmus W. Licht, Anne Hvenegaard Lund, Blazej Misiak, Patryk Piotrowski, Scott Monteith, Rodrigo Munoz, Takako Nakanotani, René E. Nielsen, Claire O’Donovan, Yasushi Okamura, Yamima Osher, Andreas Reif, Philipp Ritter, Janusz K. Rybakowski, Kemal Sagduyu, Brett Sawchuk, Elon Schwartz, Ângela Miranda Scippa, Claire Slaney, Ahmad Hatim Sulaiman, Kirsi Suominen, Aleksandra Suwalska, Peter Tam, Yoshitaka Tatebayashi, Leonardo Tondo, Eduard Vieta, Maj Vinberg, Biju Viswanath, Julia Volkert, Mark Zetin, Iñaki Zorrilla, Peter C. Whybrow, Michael Bauer

**Affiliations:** 1 Department of Psychiatry and Psychotherapy, University Hospital Carl Gustav Carus, Technische Universität Dresden, Dresden, Germany; 2 ChronoRecord Association, Fullerton, CA USA; 3 Department of Psychiatry, Dalhousie University, Halifax, Nova Scotia Canada; 4 Unit of Clinical Pharmacology, University Hospital of Cagliari, Cagliari, Italy; 5 Discipline of Psychiatry, School of Medicine, University of Adelaide, Adelaide, SA Australia; 6 IMPACT Strategic Research Centre, School of Medicine, Deakin University, Geelong, VIC Australia; 7 University Hospital Geelong, Barwon Health, Geelong, VIC Australia; 8 Department of Psychiatry, The University of Melbourne, Parkville, VIC Australia; 9 Florey Institute of Neuroscience and Mental Health, Parkville, VIC Australia; 10 Orygen Youth Health Research Centre, Parkville, VIC Australia; 11 Department of Psychiatry, Faculty of Health Sciences, Ben Gurion University of the Negev; Beer Sheva Mental Health Center, Beer Sheva, Israel; 12 Department of Psychiatry, University of Oxford, Warneford Hospital, Oxford, UK; 13 Section of Neurosciences and Clinical Pharmacology, Department of Biomedical Sciences, University of Cagliari, Sardinia, Italy; 14 Department of Molecular Medicine, Department of Mental Health (DAI), University of Siena, University of Siena Medical Center (AOUS), Siena, Italy; 15 Department of General Adult Psychiatry, Castle Peak Hospital, Hong Kong, China; 16 AP-HP, Hôpitaux Universitaires Henri-Mondor, Créteil, France; 17 Bipolar Disorder Research Program, Department of Psychiatry, University of São Paulo Medical School, São Paulo, Brazil; 18 Department of Psychiatry, University of Calgary, Calgary, Canada; 19 AP-HP, Hôpitaux Universitaires Henri-Mondor, INSERM U955 (IMRB), Université Paris Est, Créteil, France; 20 Department of Affective Disorders, Q, Mood Disorders Research Unit, Aarhus University Hospital, Aarhus, Denmark; 21 Department of Psychiatry, University Hospital of Alava, University of the Basque Country, CIBERSAM, Vitoria, Spain; 22 Mood Disorders Center of Ottawa, Ottawa, Canada; 23 Department of Psychiatry, University of Toronto, Ontario, Canada; 24 Department of Psychiatry, Tokyo Metropolitan Matsuzawa Hospital, Setagaya, Tokyo Japan; 25 Department of Psychology & Aston Brain Centre, Aston University, Birmingham, UK; 26 Institut Pasteur, Unité Perception et Mémoire, 75015 Paris, France; 27 Bipolar Disorders Program, Hospital Clinic, University of Barcelona, IDIBAPS, CIBERSAM, Barcelona, Catalonia Spain; 28 Department of Clinical Psychology, NIMHANS, Bangalore, 560029 India; 29 Leicestershire Partnership NHS Trust, Leicester, UK; 30 Department of Psychiatry, Kowloon Hospital, Hong Kong, China; 31 Aalborg University Hospital, Psychiatry, Aalborg, Denmark; 32 Department of Clinical Medicine, Aalborg University, Aalborg, Denmark; 33 Department of Psychiatry, Wroclaw Medical University, Wroclaw, Poland; 34 Michigan State University College of Human Medicine, Traverse City Campus, Traverse City, MI USA; 35 Department of Psychiatry, University of California San Diego, San Diego, CA USA; 36 Affective Disorders Research Project, Tokyo Metropolitan Institute of Medical Science, Setagaya, Tokyo Japan; 37 Department of Psychiatry, Psychosomatic Medicine and Psychotherapy, University Hospital Frankfurt, Goethe-University, Frankfurt am Main, Germany; 38 Department of Adult Psychiatry, Poznan University of Medical Sciences,, Poznan, Poland; 39 Department of Psychiatry, University of Missouri Kansas City School of Medicine, Kansas City, MO USA; 40 Croton on Hudson, NY USA; 41 Department of Neuroscience and Mental Health, Federal University of Bahia, Salvador, Brazil; 42 Department of Psychological Medicine, Faculty of Medicine, University of Malaya, Kuala Lumpur, Malaysia; 43 City of Helsinki, Department of Social Services and Health Care, Psychiatry, Helsinki, Finland; 44 Department of Psychiatry, Department of Medicine, University of Hong Kong, Hong Kong, China; 45 Harvard Medical School-McLean Hospital, Boston, MA USA; 46 Lucio Bini Center, Cagliari e Roma, Italy; 47 Psychiatric Center Copenhagen, Copenhagen, Denmark; 48 Department of Psychiatry, NIMHANS, Bangalore, 560029 India; 49 Department of Psychology, Chapman University, Orange, CA USA; 50 Department of Psychiatry and Biobehavioral Sciences, Semel Institute for Neuroscience and Human Behavior, University of California Los Angeles (UCLA), Los Angeles, CA USA

## Erratum to: Int J Bipolar Disord (2016) 4:1 DOI 10.1186/s40345-016-0058-0

Unfortunately, the original version of this article [1] contained an error. The authoriship was included incorrectly. Jörn Conell and Rita Bauer should have been listed as joint first authors as seen above.
