# Supplementary material for: Online information seeking by patients with bipolar disorder: results from an international multisite survey
Source: Int J Bipolar Disord. 2016 Aug 24;4:17. doi: 10.1186/s40345-016-0058-0 (PMC4995194; doi:10.1186/s40345-016-0058-0)
Supplement: Supplementary file 1 — Additional file 1. Questionnaire: Information seeking in bipolar disorder. [file 40345_2016_58_MOESM1_ESM.doc]

**Information seeking in bipolar disorder**

**Questions To Be Filled Out By The Physician**

Site: _______

Patient Code Number: _______

Date: _______________

1. Bipolar disorder subtype

□ BP I

□ BP II

□ BP NOS

3. Age of onset of bipolar disorder (first episode): _______

4. Years of education patient completed: _______

**Questions To Be Filled Out By The Patient**

**Thank you for participating in this international study of how patients with bipolar disorder find information on their illness. The study results will help us provide better information about bipolar disorder. Your contribution is appreciated.**

1. Age: ________

2. Sex:

□ Male

□ Female

3. What type of area do you live in? *(Check one).*

□ Urban

□ Suburban

□ Rural

4. What best describes your employment status? *(Check one).*

□ Employed full time

□ Employed part time

□ Student full time

□ Unemployed

□ Receive disability payment

□ Retired

□ Not in work force

5. What is your marital status? *(Check one).*

□ Single

□ Married

□ Living with a partner

□ Divorced

□ Separated

□ Widowed

6. Which income group would you say you belong to? *(Check one).*

□ Upper income

□ Middle income

□ Lower income

7. Do you live alone? *(Check one).*

□ Yes

□ No

8. How has your mood been over the last 6 months? *(Check one).*

□ Mostly normal

□ Mostly depressed

□ Mostly manic

□ Mostly manic and depressed

9. Has your bipolar disorder interfered with your regular activities over the last 6 months? *(Check one).*

□ Frequently

□ Sometimes

□ Rarely

□ Never

10. Are you confident in your ability to manage living with bipolar disorder? *(Check one).*

□ Very confident

□ Somewhat confident

□ Not confident at all

11. Are you confident in your ability to decide when to see a doctor for bipolar disorder? *(Check one).*

□ Very confident

□ Somewhat confident

□ Not confident at all

12. Has your physician prescribed drugs for bipolar disorder? *(Check one).*

□ Yes

□ No

**If your physician has not prescribed drugs, please skip to question 14 on page 4.**

13. How often do you take prescription drugs for bipolar disorder? *(Check one).*

□ More than 75% of time

□ 50% to 75% of time

□ 25% to 50% of time

□ Less than 25% of time

□ Never

14. Do you consult with any of these professionals to find information about bipolar disorder? (*Check all that apply*).

□ Psychiatrist

□ Primary care doctor

□ Psychologist

□ Counselor or therapist

□ Spiritual or religious counselor

□ Alternative medicine practitioner

□ Pharmacist

□ Other _______________

15. Have you ever attended any patient support groups, educational classes or group therapy, or received individual psychotherapy for bipolar disorder? *(Check one).*

□ Yes

□ No

16. Do you use any of these resources to obtain information about bipolar disorder?

(*Check all that apply*).

□ Friends or family

□ Other people with bipolar disorder

□ Newspaper or magazine articles

□ Volunteer telephone helplines

□ Television or radio programs

□ Pamphlets or handouts from doctor

□ Books

□ Internet

□ Other _______________

17. Do you use the Internet? *(Check one).*

□ Yes

□ No

**If you do use the Internet, please skip to question 19 on Page 6.**

18. If you do not use the Internet, why not? *(Check all that apply).*

□ Lack access to a computer

□ Never learned how to use the Internet

□ It's too expensive

□ Anxious about the use of computers

□ Computer breaks too often

□ It's too slow

□ It's too technical and too hard.

□ Friends or family go online for you

□ Other _______________

**Thank you. You are finished with the survey!**

19. Do you use the Internet to find out about bipolar disorder? *(Check one).*

□ Yes

□ No

**If you use the Internet to find information about bipolar disorder, please skip to question 21 on Page 7.**

20. Why don't you use the Internet to find information about bipolar disorder? *(Check all that apply).*

□ Prefer to rely on information from a doctor

□ Too much information

□ Afraid of Internet addiction

□ Distrust information on the Internet

□ Too hard to concentrate

□ There's nothing of interest on the Internet

□ Privacy or confidentiality concerns

□ Don't know how to search the Internet

□ Other _______________

**Thank you. You are finished with the survey!**

21. Most frequently, how do you access the Internet to find information about bipolar disorder? *(Check one).*

□ Computer at home

□ Computer at school or work

□ Computer in library

□ Computer in other public access such as Cybercafe

□ Computer at home of friend or family member

□ Smartphone

□ Tablet

□ Other _______________

22. How would you rate your Internet search skills? *(Check one).*

□ Basic

□ Intermediate

□ Expert

23. The last time you used the Internet to find information about bipolar disorder, how did you start looking? *(Check one).*

□ Search engine such as Google or Yahoo

□ Medical search engine such as MedlinePlus

□ Medical web site such as WebMD

□ Specific site on mental illness or bipolar disorder

□ Social network site such as Facebook

□ Encyclopedia site such as Wikipedia

□ Other _______________

24. When you search for information online about bipolar disorder, do you find what you are looking for? *(Check one).*

□ Always

□ Most of the time

□ About half the time

□ Less than half the time

□ Rarely

□ Never

25. Have you searched the Internet for information about any of these topics relating to bipolar disorder? *(Check all that apply).*

□ Prescription drug information

□ Symptoms

□ General course of illness

□ Coping strategies

□ Psychotherapy options

□ Patient support groups

□ Alternative and experimental treatments

□ Other _______________

26. Have you searched the Internet for information about any of these topics relating to getting treatment for bipolar disorder? *(Check all that apply).*

□ Clinic hours, location and directions

□ Physician or therapist credentials

□ Physician or therapist ratings

□ Financial support available

□ Legal issues

□ Purchase of prescription drugs (online pharmacy)

□ Other _______________

27. How frequently do you search for information about bipolar disorder on the Internet? *(Check one).*

□ Daily

□ Weekly

□ Monthly

□ Couple times a year

28. Why do you search for information on bipolar disorder on the Internet? (*Check all that apply).*

□ Do not obtain enough information from my doctor

□ Do not understand the information from my doctor

□ To learn anonymously

□ Side effects from my prescription drugs

□ To double check treatment advice

□ Not convinced my diagnosis is correct

□ Need help coping with the illness

□ To learn about non-drug therapies

□ To find another doctor

□ To save money

□ Other _______________

29. What are your favorite online sources of information about bipolar disorder? (*Check all that apply.)*

□ Government-sponsored sites such as MedlinePlus

□ Health web pages such as WebMD

□ Specific sites on mental health or bipolar disorder

□ Medical journals

□ Academic/research institutions

□ Pharmaceutical companies

□ Patient support groups, chats or forums

□ Online encyclopedia such as Wikipedia

□ Individual physicians

□ Alternative medicine sites

□ Other _______________

30. Do you discuss the information you learned online about bipolar disorder with your doctor? *(Check one).*

□ Frequently

□ Sometimes

□ Rarely

□ Never

31. Does the information you learned online help you to cope with bipolar disorder? *(Check one).*

□ Frequently

□ Sometimes

□ Rarely

□ Never

32. Do you attempt to verify the information you find online about bipolar disorder? *(Check one).*

□ Yes

□ No

**If you do not attempt to verify the information, please skip to question 34.**

33. How do you attempt to verify the information you find online about bipolar disorder? (*Check all that apply).*

□ Discuss with my doctor

□ Discuss with family and friends

□ Compare information on multiple web sites

□ Read "about us" pages

□ Look for endorsement from government or professional society

□ Look for pharmaceutical company sponsor

□ Look for medical related advertising on site

□ Look for content approval markers such as from HON (Health on the Net Foundation)

□ Other _______________

34. How concerned are you about privacy and confidentiality when you search the Internet for information on bipolar disorder? *(Check one).*

□ Very concerned

□ Moderately concerned

□ Slightly concerned

□ Not concerned

35. Do you read or participate in online patient support groups, chats or forums for bipolar disorder? *(Check one).*

□ Yes

□ No

**If you read or participate in online support groups, please skip to question 36 on page 12.**

**Thank you. You are finished with the survey!**

36. Why do you participate in online bipolar disorder support groups, chats or forums? (*Check all that apply.)*

□ 7/24 access to others with bipolar disorder

□ Help with emotional aspects of illness

□ To talk about lifestyle issues such as diet and exercise

□ For information on alternative treatments

□ For recommendations about doctors

□ To find moral support without burdening family

□ For general chat

□ Other _______________

37. How frequently do you read online patient support groups, chats or forums for bipolar disorder? *(Check one).*

□ Daily

□ Weekly

□ Monthly

□ Couple times a year

38. How useful is the information in online patient support groups, chats or forums for bipolar disorder? *(Check one).*

□ Very useful

□ Somewhat useful

□ Not too useful

□ Not useful at all

39. Do online patient support groups, chats or forums help you to cope with bipolar disorder? *(Check one).*

□ Frequently

□ Sometimes

□ Rarely

□ Never

**Thank you. You are finished with the survey!**
